# CFD Simulation of Flow Behavior and Apparent Viscosity Distribution of Polymer Solution in a Rotating Packed Bed

**DOI:** 10.3390/polym18202553

**Published:** 2026-10-16

**Authors:** Cheng Fu, Zhi-Yao Wang, Xue-Song Yin, Yi-Ping Zhang, Rui Zhu, Wei Zhang, Bin Huang

**Affiliations:** 1 School of Petroleum Engineering, Chongqing University of Science and Technology, Chongqing 401331, China; 2 Chengdu Natural Gas Chemical General Plant, PetroChina Southwest Oil & Gasfield Company, Chengdu 610072, China; 3 Chongqing Institute of Unconventional Oil and Gas Development, Chongqing University of Science and Technology, Chongqing 401331, China

**Keywords:** polymer dissolution, CFD simulation, rotating packed bed

## Abstract

High-gravity rotating packed beds enhance mass transfer via high-gravity fields, where the flow and viscosity distribution of polymer solutions govern dissolution efficiency and equipment compactness. Understanding these behaviors is essential for optimizing polymer dissolution in offshore polymer flooding, yet experimental methods are hindered by poor light transmittance and observational blind spots, making it difficult to capture the dynamic dispersion and evolution of polymer solutions within the wire mesh packing. To address this issue, a two-dimensional computational model was constructed by combining the VOF multiphase model with the Carreau–Yassuda viscosity model, accounting for the shear-thinning characteristics and viscosity variations of the polymer solution. The effects of zero-shear viscosity, rotational speed, and inlet velocity on flow morphology, aggregation, and apparent viscosity distribution were systematically examined. Results show the following: (1) Increasing rotational speed from 300 to 900 rpm promotes the transition from film-dominated to droplet-dominated flow, increasing zero-shear viscosity from 59 to 348 mPa·s suppresses dispersion and forms coexisting films and droplets, and increasing inlet velocity from 1.74 to 3.17 m/s drives the transition from droplet-dominated to film-dominated flow. (2) Increasing rotational speed reduces apparent viscosity by up to 30% and improves distribution uniformity, with a turning point near 1000 rpm; inlet velocity has limited effects, with radial distribution higher in the inner region. (3) The Carreau–Yassuda model reveals a shear-thinning effect, where high rotational speed mitigates the influence of high zero-shear viscosity, and the combination of high rotational speed and high inlet velocity synergistically enhances dispersion. These findings provide a theoretical basis for optimizing polymer dissolution in high-gravity carbon capture processes.

## 1. Introduction

With the continuous expansion of oil reservoir development in offshore fields such as the Bohai Sea and the South China Sea, polymer flooding technology, as an effective means of enhanced oil recovery, is progressively being extended to offshore oilfield applications [1,2,3]. However, the unique operating conditions on offshore platforms pose multiple technical challenges for polymer injection systems, primarily manifested in high injection rate requirements per well, limited platform deck space, and prolonged polymer dissolution time, all of which impose higher performance demands on polymer dissolution equipment. Conventional dissolution devices commonly suffer from low maturation efficiency, non-uniform dissolution, a large footprint, and high energy consumption, which have become critical bottlenecks restricting the efficiency of polymer injection [4,5]. Therefore, there is an urgent need to introduce novel technologies to accelerate the polymer dissolution process and reduce equipment size, thereby effectively addressing the aforementioned constraints.

As a significant breakthrough in the field of process intensification, high-gravity technology, represented by its core equipment, the rotating packed bed (RPB), generates a high-gravity field through high-speed rotation, which can significantly enhance mass transfer efficiency [6,7,8]. The application of RPB to polymer dissolution processes is expected to achieve a substantial reduction in dissolution time while minimizing mechanical damage to polymer molecular chains, along with a reduced energy consumption and equipment footprint, providing a novel solution for overcoming the current technical bottlenecks in polymer flooding technology [9].

In a RPB, the liquid phase exhibits diverse flow states and dispersion characteristics under the combined effects of the high-gravity field and the packing structure. Different dispersion mechanisms and flow pattern evolutions directly influence the mixing and mass transfer efficiency, thereby affecting the polymer dissolution process. Early visualization studies [10] revealed that the liquid phase in metal wire mesh packings primarily exhibits three dispersion forms: (1) orifice flow, where the liquid forms continuous channels along the packing pores, typically occurring at low rotational speeds; (2) droplet flow, where the liquid film breaks into discrete droplets under centrifugal force, dominating the medium to high rotational speed range; and (3) liquid film flow, where the liquid covers the packing surface as a thin film, often observed under high-viscosity fluid conditions. Guo et al. [11] found that the liquid film thickness on metal foam packings is significantly greater than that on metal wire mesh packings, due to differences in skeleton structure. Yang et al. [12] further confirmed that the liquid holdup in nickel foam packings is higher than that in metal wire mesh packings, which may be attributed to the larger specific surface area of the nickel foam. Xie et al. [13,14], Yang et al. [15], Ouyang et al. [16], and Lu et al. [17,18] employed CFD simulations to directly reveal the liquid flow states within the packing zone of the RPB, clearly capturing the changes in liquid flow behavior and obtaining quantitative data on liquid holdup in the packing zone. The simulation results indicate that increasing rotational speed breaks the liquid into finer micro-elements, with the flow pattern gradually transitioning from film-dominated to droplet flow, while increasing liquid flow rate significantly enhances the turbulent intensity of the liquid phase. Lu et al. [19] investigated the dispersion performance of stainless steel wire mesh packings on liquids and found that surface characteristics significantly affect dispersion efficiency; hydrophobically modified stainless steel wire mesh can reduce the average droplet size. Xu et al. [20] conducted liquid jet impact experiments on a single-layer stainless steel wire mesh to reveal the influence mechanism of the wire structure: the longitudinal wires promote liquid breakup through shear action, while the transverse wires induce circumferential distribution through carrying action, and the synergistic effect of the two determines the liquid flow state.

Although numerous scholars have conducted systematic studies on the dispersion behavior and flow pattern evolution of the liquid phase in the packing zone of rotating packed beds using visualization experiments and CFD simulations, most of these investigations have focused on conventional Newtonian fluid systems. However, in actual oilfield development, inelastic non-Newtonian fluids, represented by polymer solutions, are increasingly being applied, while research on the flow and dispersion characteristics of such fluids in rotating packed beds remains relatively scarce. This gap is significant because the shear-thinning behavior of polymer solutions fundamentally alters the flow morphology and dispersion mechanisms, making the dispersion and flow pattern evolution laws derived from Newtonian fluid systems not directly applicable to non-Newtonian systems. Consequently, the design and optimization of related process intensification equipment are hindered by the lack of a reliable theoretical basis for predicting non-Newtonian flow behavior in high-gravity fields. Moreover, existing CFD models rarely incorporate non-Newtonian viscosity models such as the Carreau–Yassuda model to describe polymer solution behavior under high shear rates, further limiting the accuracy of numerical predictions.

To address these knowledge gaps, the present study established a VOF multiphase flow model incorporating the Carreau–Yassuda viscosity model. The wire mesh packing was simplified as a two-dimensional wire array, and the effects of zero-shear viscosity of the polymer solution, rotor rotational speed, and inlet velocity on the liquid flow morphology, aggregation state, and spatial distribution of apparent viscosity were systematically investigated. Particular emphasis was placed on analyzing the flow pattern transitions and apparent viscosity distribution characteristics induced by shear-thinning effects in the packing zone, revealing the compound flow pattern evolution mechanisms of inelastic non-Newtonian fluids in high-gravity rotating packed beds. These findings provide a theoretical basis for the design and optimization of polymer dissolution equipment in offshore oilfield applications.

## 2. CFD Simulation

### 2.1. Geometry Model

**Figure 1:**
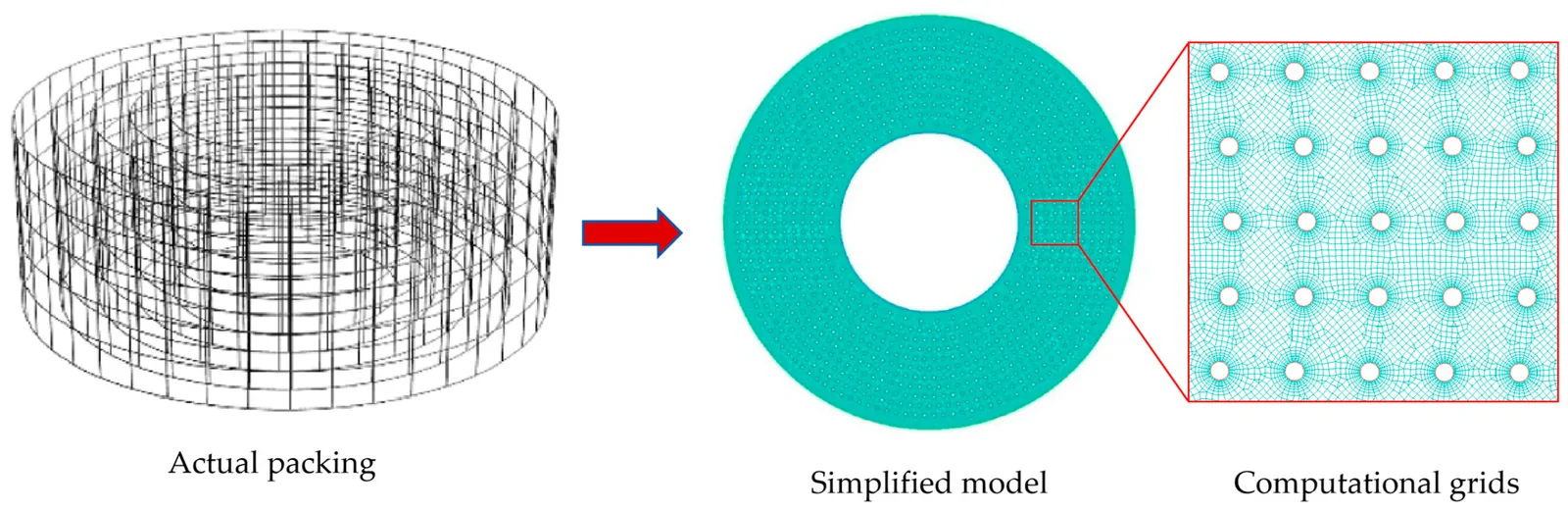
Geometric model of RPB packing.

Due to the complex internal packing structure of the RPB, a reasonable simplification of the packing geometry was made in this study, following the treatment methods reported in the literature. According to previous findings [20], the transverse wires primarily serve to carry the liquid, while the longitudinal wires mainly exert shear effects. Therefore, the carrying effect of the transverse wires can be neglected, and the longitudinal wires are retained as the representative structural elements of the packing. As shown in Figure 1, a two-dimensional CFD model of the entire RPB was established using cylindrical longitudinal wires as the computational objects, with each packing ring containing a finite number of cylindrical wires. The geometric dimensions of the computational domain are as follows: the inner diameter is 80 mm, the outer diameter is 160 mm, the diameter of the solution inlet nozzle is 2 mm, the diameter of each cylindrical wire is 0.6 mm, the circumferential spacing between adjacent wires is 3 mm, and the radial spacing is 2 mm. A total of 20 rings of wire mesh packing are arranged, and the overall porosity of the packing layer is 95%.

### 2.2. Computational Grids

**Figure 2:**
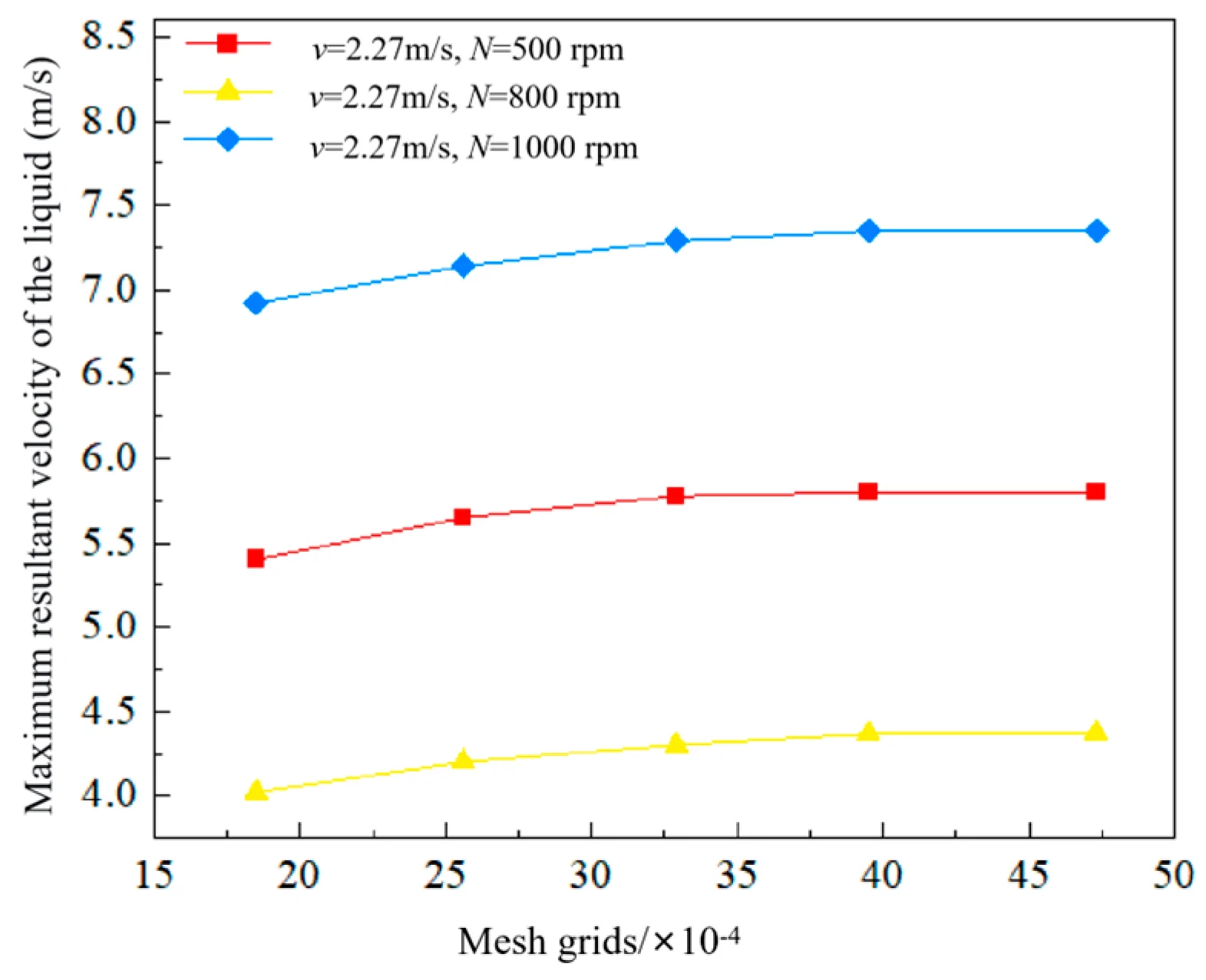
Mesh grid independence.

To reduce computational cost, the two-dimensional model does not include the inner and outer cavity regions; the computational grid is established only within the rotating packing zone. An unstructured meshing method is adopted, with an average mesh quality exceeding 0.8. The resultant velocity of the tangential and circumferential velocities of the solution in the packed bed is selected as the characteristic velocity, and the maximum resultant velocity is used as the criterion for grid independence verification. Five sets of grids with element counts of 185,240, 256,842, 329,770, 395,405, and 473,521 were tested. Figure 2 presents the grid independence verification results. It can be observed that when the grid number reaches approximately 320,000, further increases in grid count no longer cause significant changes in the resultant velocity, indicating that grid independence is achieved at this level. Therefore, the grid model with an original element count of 329,770 was adopted for subsequent simulations.

### 2.3. Mathematical Model

In this study, an unsteady simulation approach was adopted to capture the flow process of the polymer solution within the packing zone. The liquid flow in the region was treated as transient, incompressible, and isothermal, with heat sources neglected and no heat or mass transfer involved. Consequently, only the continuity and momentum equations were solved. To capture the complex gas–liquid interface evolution in the RPB, the volume of fluid (VOF) model proposed by Hirt and Nichols was employed [21,22,23]. The constructed RPB model does not include the inner and outer cavity regions, but only covers the rotating zone of the packing. The corresponding continuity and momentum equations can be expressed as follows:

(1) $$\frac{\partial }{\partial t}(\alpha_{l}\rho_{l})+\nabla \cdot (\alpha_{l}\rho_{l}\overset{\to }{v_{\mathrm{lr}}})=0$$

(2) $$\frac{\partial }{\partial t}\left(\rho \overset{\to }{v_{r}}\right)+\nabla \cdot \left(\rho \overset{\to }{v_{r}}\overset{\to }{v_{r}}\right)+\rho \left(2\overset{\to }{\omega }\times \overset{\to }{v_{r}}+\overset{\to }{\omega }\times \overset{\to }{\omega }\times \overset{\to }{r}\right)=-\nabla p+\nabla \cdot [\mu (\nabla \overset{\to }{v_{r}}+\nabla \overset{\to }{v_{r}^{T}})]+\overset{\to }{F_{\mathrm{vol}}}$$

where and $\left(\overset{\to }{\omega }\times \overset{\to }{\omega }\times \overset{\to }{r}\right)$ represent the Coriolis acceleration and the centrifugal acceleration, respectively, in m/s^2^. The term α_l_ denotes the volume fraction of the liquid phase, ρ_l_ is the density of the polymer solution in kg/m^3^, t is the elapsed time in seconds, and $\overset{\to }{v_{\mathrm{lr}}}$ is the relative velocity of the liquid phase in m/s.

The volume fraction is calculated by the following equation:

α_g_ = 1 − α_l_ (3)

When α_l_ = 1, the grid cell is completely filled with the liquid phase; when α_l_ = 0 the cell is entirely occupied by the gas phase; when 0 < α_l_ < 1, the cell contains both gas and liquid phases, indicating the presence of a gas–liquid interface.

The volumetric force term F_vol_ is computed using the Continuum Surface Force (CSF) model proposed by Brackbill et al. [24]. This model is a classical approach for accounting for surface tension effects. Its core principle is to convert the surface tension acting at the interface into an equivalent volumetric force acting on the fluid near the interface, thereby avoiding the need for direct tracking of the interface boundary conditions and facilitating the numerical simulation of gas–liquid two-phase flows within the VOF framework.

Its calculation formula is as follows:

(4) $$F_{\mathrm{vol}}=\sigma \frac{\rho k\nabla \alpha_{l}}{0.5(\rho_{g}+\rho_{l})}$$

where σ is the surface tension coefficient, and *k* is the interface curvature, which can be calculated by the following equation:

(5) $$k=\nabla \cdot \overset{\sim }{n}$$

where $\overset{\sim }{n}=n/|n|,n=\nabla \alpha_{l}$.

### 2.4. Boundary Conditions

In this work, the liquid inlet is located at the inner ring of the packing adjacent to the inner cavity region, corresponding to the inner diameter boundary of the model, where a velocity inlet boundary condition is applied. The liquid outlet is situated at the outer ring of the packing adjacent to the outer cavity region, corresponding to the outer diameter boundary of the model, where a pressure outlet boundary condition is employed. The inlet velocity at the solution nozzle is fixed in the range of 1.74 to 3.17 m/s. The packing is fixed in a rotating reference frame, with rotational speeds ranging from 300 to 1500 rpm. The nozzle moves in the opposite direction relative to the packing, and its motion trajectory is controlled by a user-defined function (UDF). Partially hydrolyzed polyacrylamide (HPAM) was selected as the model polymer solution because it is the most widely used polymer for enhanced oil recovery in offshore oilfields, particularly in the Bohai Sea and South China Sea, where polymer flooding has been increasingly applied. The physical properties of the HPAM solution are also defined through UDFs. The polymer solution is a typical non-Newtonian fluid, exhibiting a nonlinear relationship between shear stress and strain rate, with pronounced shear-thinning behavior. To describe the variation in its viscosity with shear rate, the Carreau–Yassuda model is adopted in this study to predict the apparent viscosity of the polymer solution. This is because the Carreau–Yassuda model can accurately describe the viscosity transition from the zero-shear Newtonian plateau to the power-law shear-thinning region, and it has been widely adopted for modeling polymer solutions in enhanced oil recovery applications. The basic expression of the model is as follows [25]:

(6) $$\eta \left(\dot{\gamma }\right)=\eta_{\infty }+\frac{\eta_{0}-\eta_{\infty }}{{\left[1+{\left(\lambda \dot{\gamma }\right)}^{\alpha }\right]}^{\frac{1-n}{\alpha }}}$$

where $\eta \left(\dot{\gamma }\right)$ is the apparent viscosity at a given shear rate, Pa·s, η_0_ is the zero-shear Newtonian viscosity, and η_∞_ is the limiting viscosity at infinite shear rate. The parameter λ is the relaxation time (s), which characterizes the critical shear rate at which the fluid transitions from Newtonian to power-law behavior. The parameter *α* is a transition coefficient that controls the curvature of the transition from the zero-shear Newtonian plateau to the shear-thinning power-law region. The parameter n is the power-law index, which governs the intensity of shear-thinning behavior. When 0 < n < 1, the fluid exhibits shear-thinning power-law behavior; when n = 1, the fluid behaves as a Newtonian fluid. For a typical HPAM solution with a concentration of 0.15 wt% [26], the model parameters are zero-shear viscosity *η*_0_ = 151 mPa·s, infinite-shear viscosity *η*_∞_ = 1 mPa·s, relaxation time λ = 0.05 s, transition parameter α = 2, and power-law index n = 0.6.

### 2.5. Simulation Implementation

In this study, the flow and viscosity distributions of polymer solution were performed using the ANSYS Fluent 2022 software in a double-precision mode. The VOF method was selected because it provides good mass conservation and can naturally capture complex interfacial topological changes such as film rupture, ligament stretching, and droplet breakup, which are essential for describing the dispersion behavior of polymer solutions in a rotating packed bed. A steady-state pressure-based solver was employed for numerical computations, with the coupling of the pressure and velocity fields achieved using the SIMPLEC algorithm. The spatial discretization of the governing equations was performed using the finite volume method, in which the momentum equation was discretized with a second-order upwind scheme to enhance computational accuracy, while the remaining equations were discretized with a first-order upwind scheme to ensure solution stability and convergence efficiency. The residuals of all physical variables converged to below 10^−3^, indicating that the computed results satisfied the established convergence criteria. This combined strategy effectively maintains numerical stability while ensuring computational accuracy, making it suitable for addressing the nonlinear fluid dynamics problems involved in the present study.

### 2.6. Model Validation

To validate the accuracy of the numerical simulation results, the liquid holdup obtained from CFD simulations under different inlet flow velocities was compared with the predicted values calculated using the empirical correlation proposed by Burns [27]. This correlation has been widely adopted by several researchers, including Xie [13,14], and Lu [17,18], for predicting liquid holdup in RPBs. The specific expression of the correlation is as follows:

(7) $$\varepsilon_{L}=0.039{\left(\frac{g}{g_{0}}\right)}^{-0.5}{\left(\frac{U}{U_{0}}\right)}^{0.6}{\left(\frac{\nu }{\nu_{0}}\right)}^{0.22}$$

where g_0_ = 100 m/s^2^, U_0_ = 0.01 m/s, ν_0_ = 10^−6^ m^2^/s^2^.

**Figure 3:**
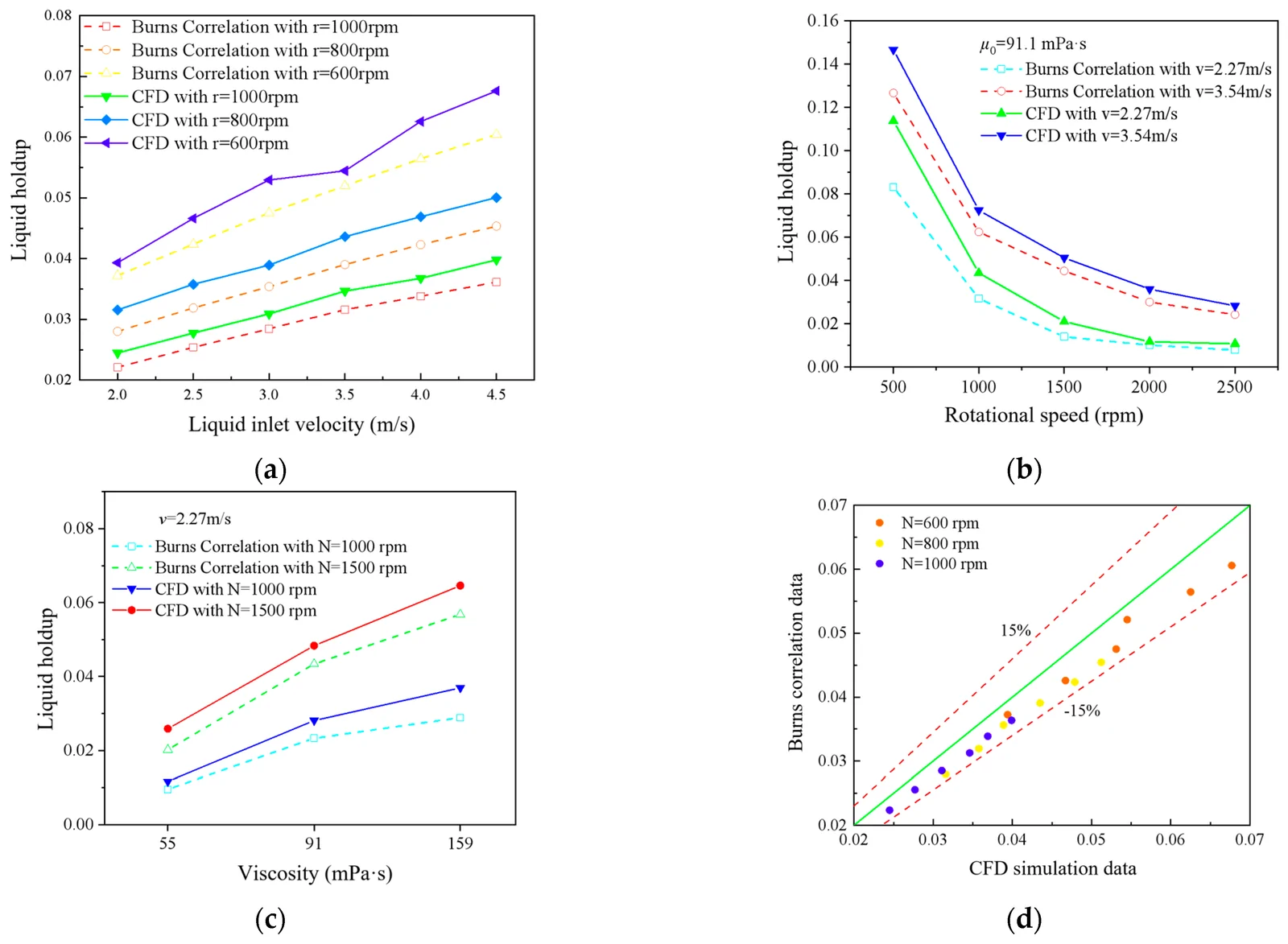
Comparison of CFD simulation results of liquid holdup with Burns predictions: (**a**) liquid inlet velocity; (**b**) rotational speed; (**c**) viscosity; and (**d**) relative error.

Figure 3 presents a comparison between the CFD simulation results of liquid holdup and the predicted values from Burns’ empirical correlation under various operating conditions, along with the corresponding diagonal error distribution. As shown in the figure, the simulation results are in good agreement with the predicted values, with relative errors generally within 15%. The liquid holdup values obtained from CFD simulations are consistently higher than those predicted by Burns’ correlation. This deviation is primarily attributed to the differences in experimental measurement methods. In Burns’ study, liquid holdup was indirectly determined using the resistivity method, which relies on the relationship between electrical resistance signals and residence time. This approach is unable to accurately capture the freely dispersed fine droplets within the packing region, resulting in an underestimation of liquid holdup in the empirical correlation.

## 3. Results and Discussion

### 3.1. Liquid Flow Pattern and Distribution Characteristics

**Figure 4:**
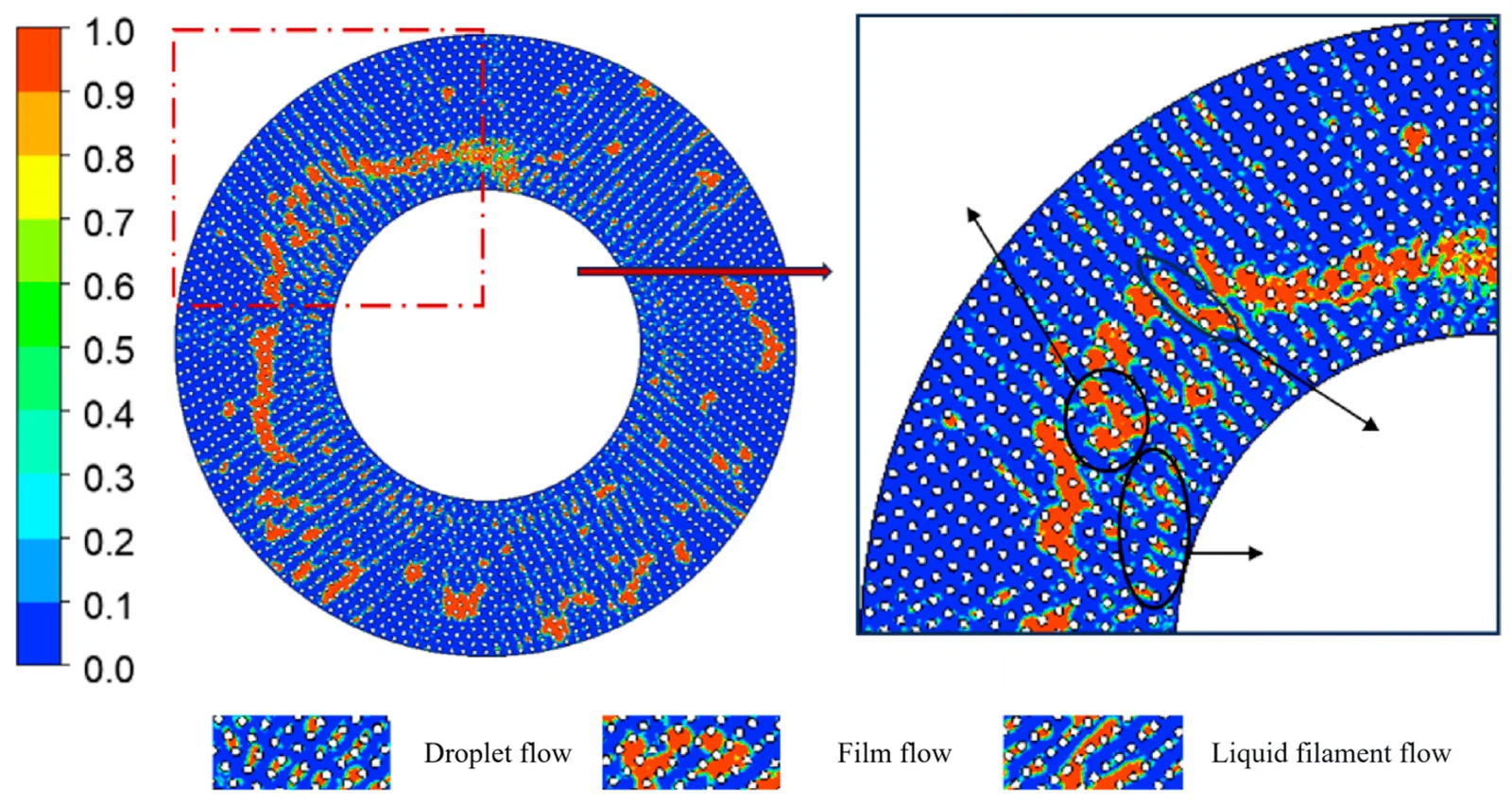
Flow pattern of polymer solution in the packing zone of the RPB.

To investigate the effects of rotational speed, inlet velocity, and zero-shear viscosity on the liquid flow behavior in the RPB packing zone, numerical simulations of the liquid phase flow morphology were conducted under various operating conditions. Figure 4 presents the simulated liquid distribution in the packing zone and its corresponding local magnified view at a rotational speed of 500 rpm, an inlet velocity of 2.27 m/s, and a solution viscosity of 55.4 mPa·s. The simulation results reveal a distinctly non-uniform liquid distribution within the packing zone, which can be categorized into three primary flow morphologies: (1) discretely distributed droplets, (2) continuously spreading liquid films, and (3) elongated liquid filaments. Among these, liquid films and droplets are the dominant flow forms. The flow pattern transitions are now attributed to the competition between centrifugal shear, which promotes film breakup into droplets, and viscous resistance, which suppresses breakup at higher initial viscosity.

#### 3.1.1. Effect of Rotational Speed

**Figure 5:**
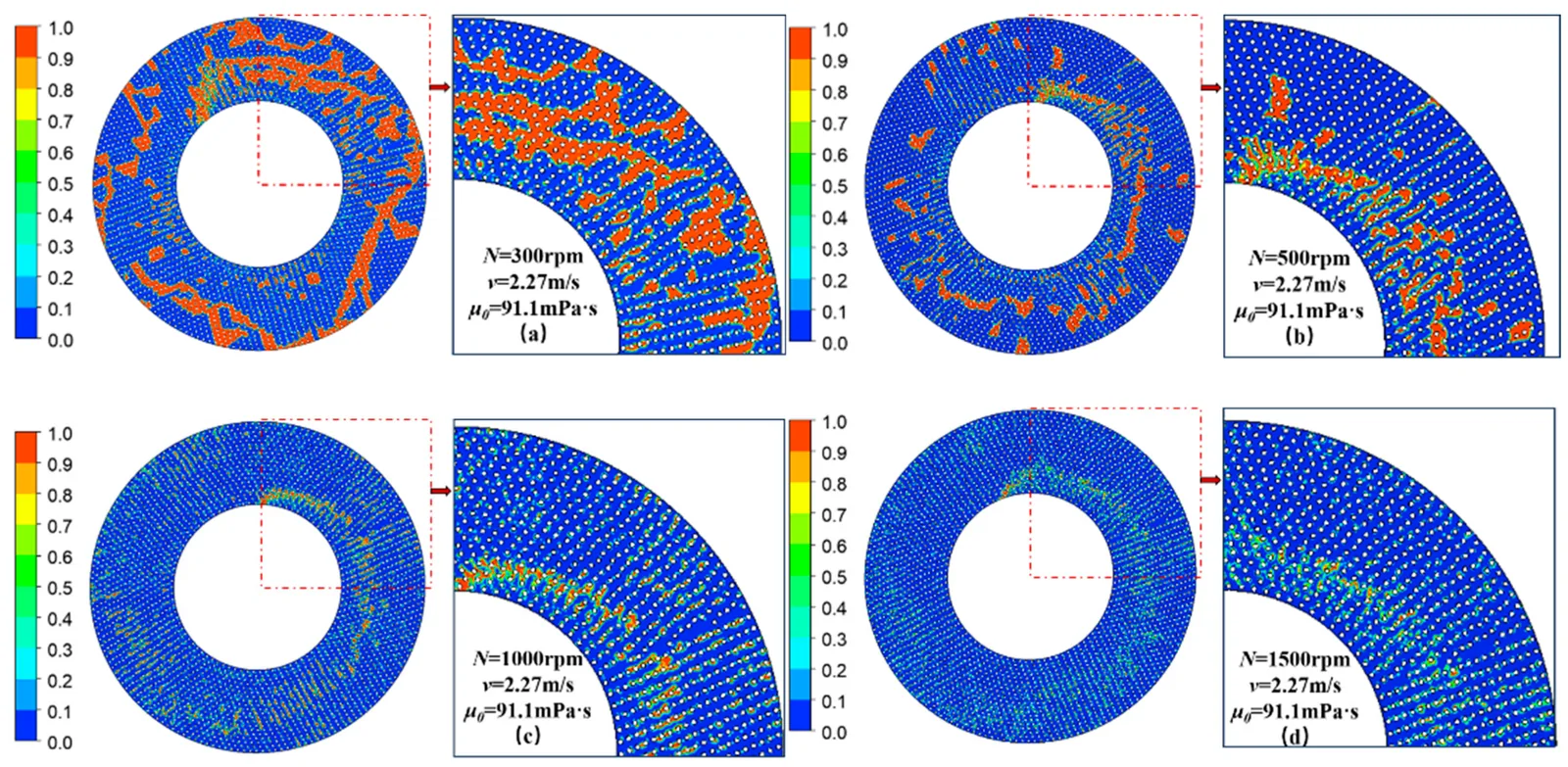
Polymer solution flow patterns in the RPB under different rotational speeds. (**a**) 300 rpm; (**b**) 500 rpm; (**c**) 1000 rpm; (**d**) 1500 rpm.

Figure 5 illustrates the influence of rotational speed on the flow pattern of the polymer solution in the RPB. It can be observed that the polymer solution initially enters the packing layer as a continuous liquid jet. Upon contacting the first few layers of wire mesh packing, the jet is rapidly sheared and broken up, primarily generating numerous irregular liquid lumps and liquid ligaments, along with a small number of dispersed droplets. Notably, these fragmented liquid elements, including droplets, ligaments, and lumps, tend to re-aggregate during subsequent motion, gradually merging into new continuous liquid phases. However, these newly formed liquid bodies are promptly re-sheared and broken up by the subsequent packing layers. This phenomenon is primarily attributed to the high molecular weight, high surface tension, and relatively high viscosity of the polymer solution, which promote liquid agglomeration. Only under certain conditions, when the fluid elements collide with each other and experience sufficient energy dissipation, can they be more readily sheared into dispersed droplets.

**Figure 6:**
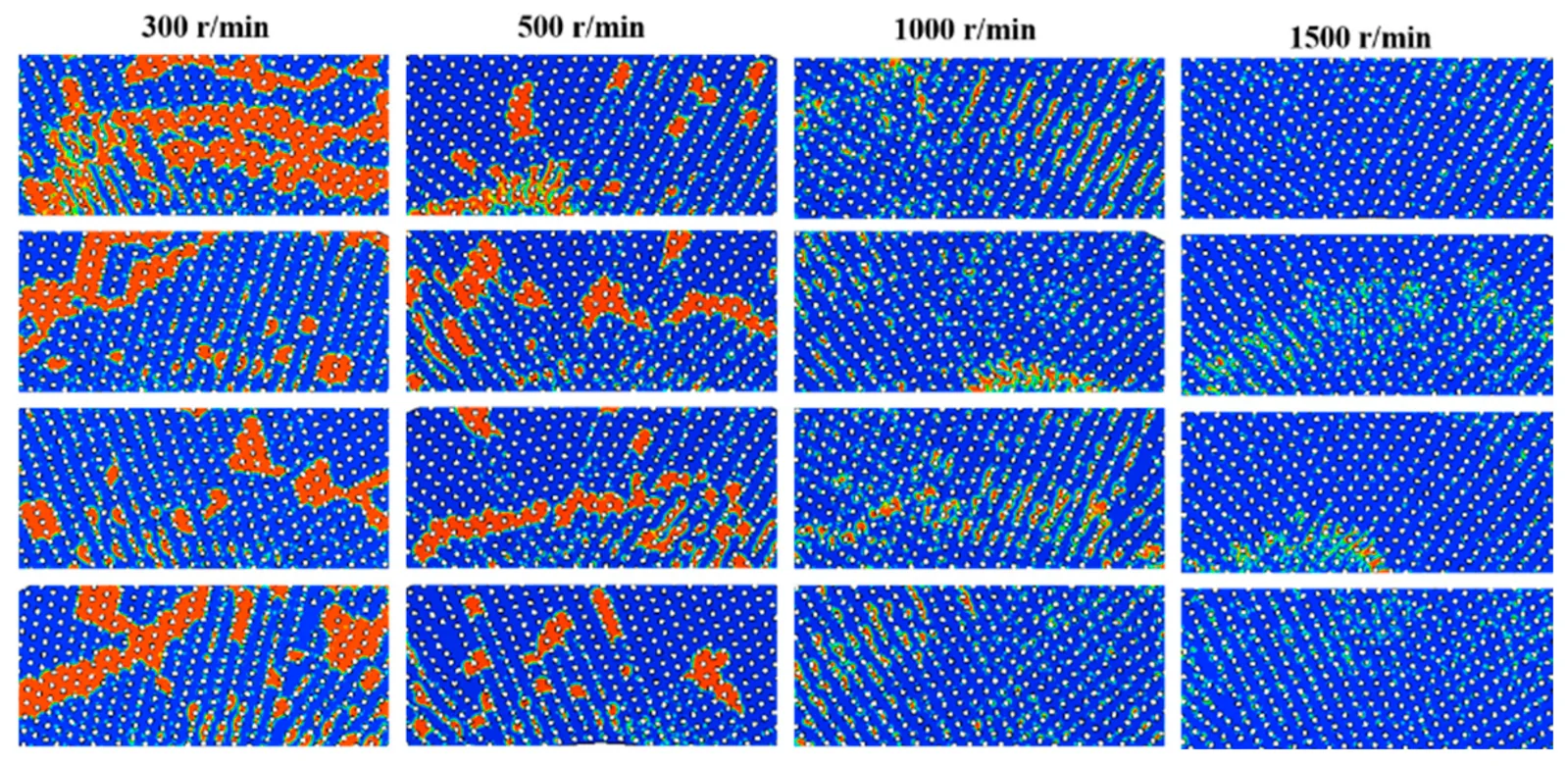
Flow pattern diagrams in packing under different rotational speeds.

Figure 6 presents magnified views of the local flow morphology. As shown in the figure, with increasing rotational speed, the tangential velocity of the packing increases, leading to enhanced shear capability, which facilitates the breakup of the liquid by the packing wires. At a low rotational speed of 300 rpm, the packing wires are unable to shear the continuous liquid into droplets, and the liquid flow pattern is predominantly of the orifice-flow type. In the medium rotational speed range of 500 to 1000 rpm, ligament-type flow is mainly observed in the packing region near the inner cavity, while the remaining packing zones are dominated by micro-droplets, liquid films, and liquid filaments. Thus, at medium rotational speeds, the liquid flow is primarily in the form of liquid films. At a high rotational speed of 1500 rpm, liquid films and filaments appear only at the inner edge of the packing, while the rest of the packing region is predominantly occupied by droplets. Therefore, under ultra-high rotational speeds, the liquid flow is mainly in the form of droplet flow.

#### 3.1.2. Effect of Zero-Shear Viscosity

**Figure 7:**
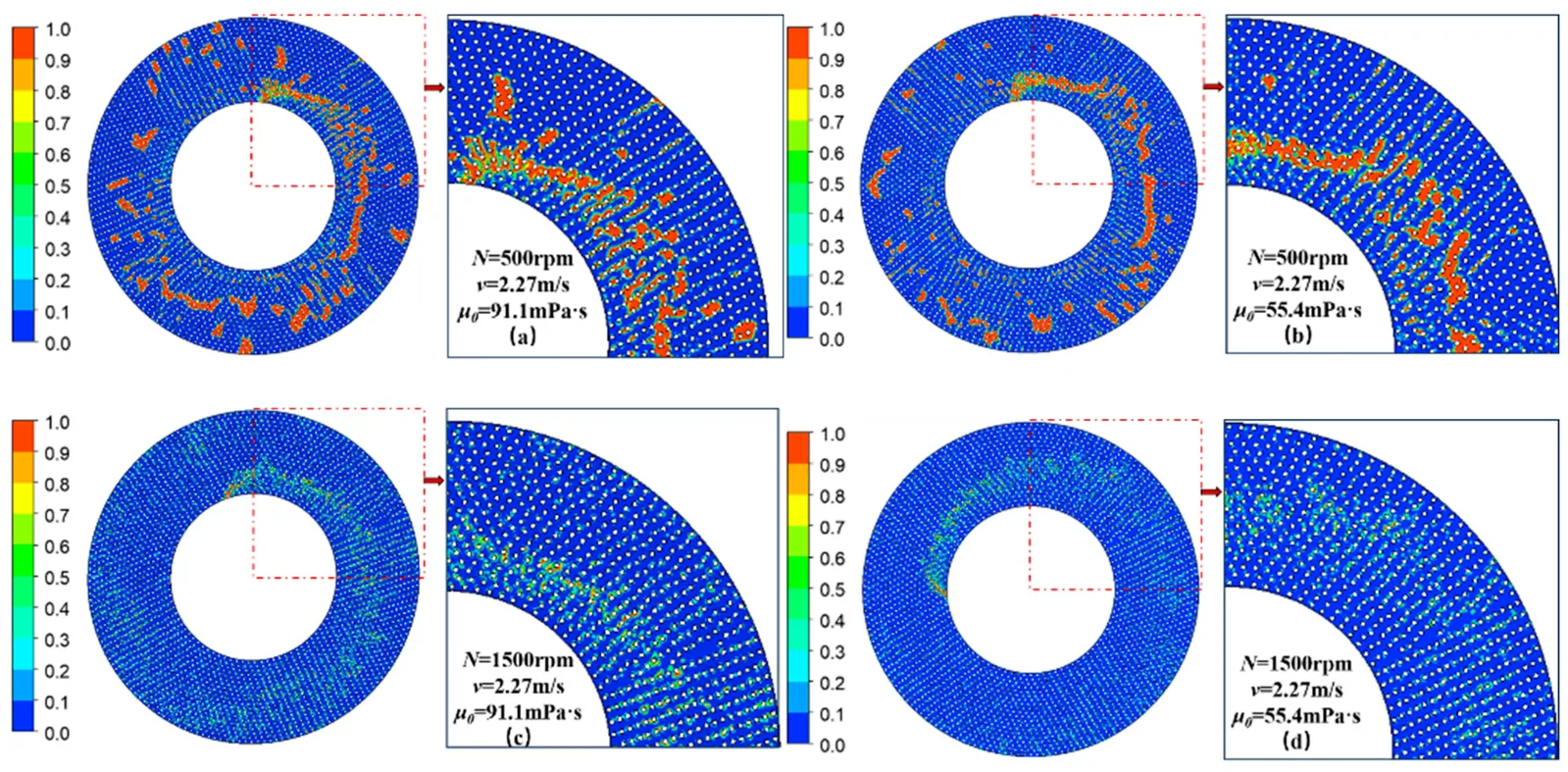
Polymer solution flow patterns in the RPB under different initial viscosities. (**a**) *N* = 500 rpm, *μ* = 91.1 mPa·s; (**b**) *N* = 500 rpm, *μ* = 55.4 mPa·s; (**c**) *N* = 1500 rpm, *μ* = 91.1 mPa·s; (**d**) *N* = 1500 rpm, *μ* = 55.4 mPa·s.

**Figure 8:**
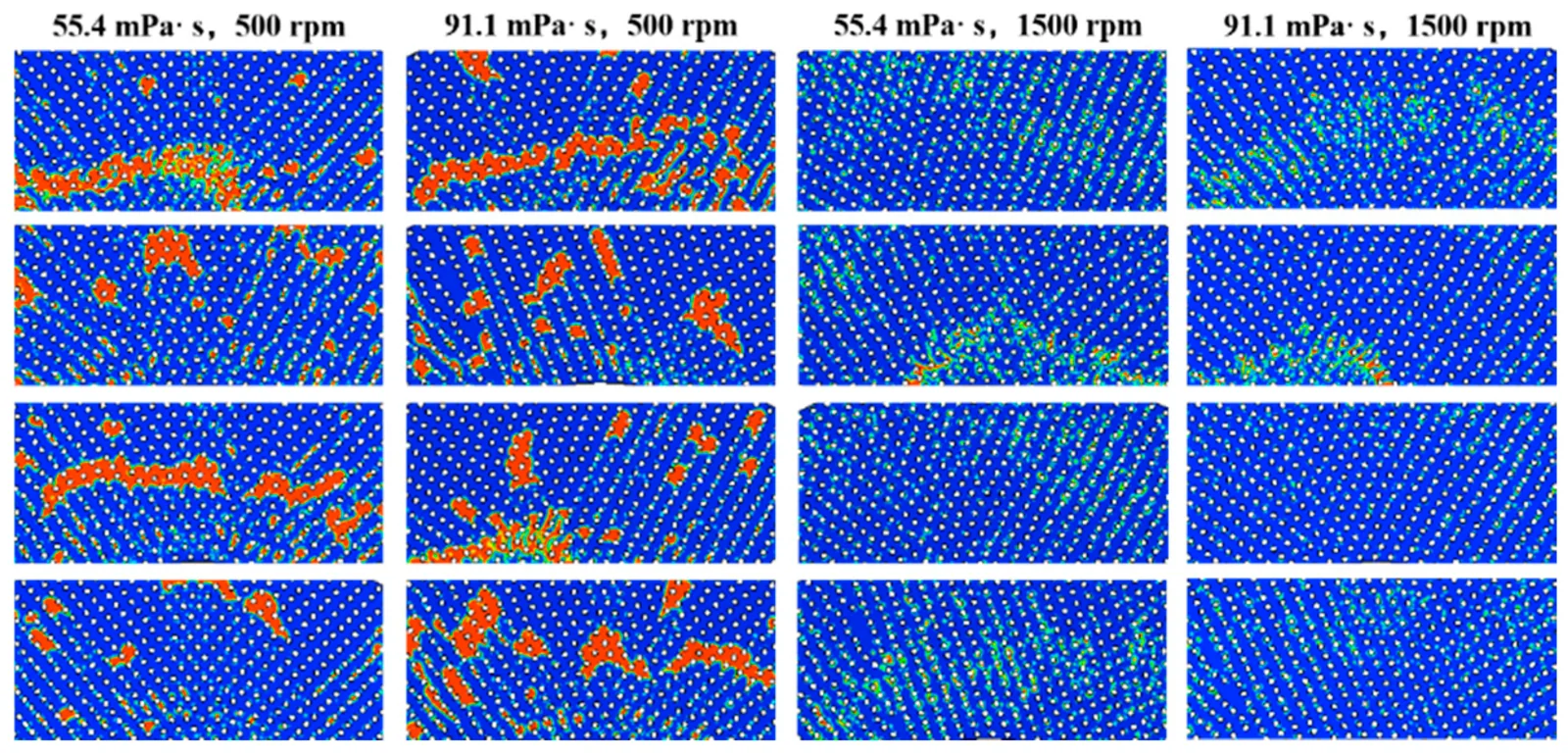
Flow pattern diagrams in packing under different initial viscosities.

Figure 7 illustrates the effect of zero-shear viscosity on the flow characteristics of the polymer solution in the RPB. It can be observed that although the diameter of liquid micro-elements does not differ significantly with varying initial viscosities, the flow velocity is noticeably reduced under high-viscosity conditions due to greater flow resistance. This velocity reduction results in a substantial increase in liquid holdup within the packing zone at higher viscosities. Figure 8 presents magnified views of the local flow morphology, allowing clearer observation of the fluid motion characteristics in specific regions. At a high rotational speed of 1500 rpm, higher zero-shear viscosity tends to promote the formation of liquid films and filaments, which subsequently break into droplets, leading to a coexistence of liquid films and dispersed droplets. In contrast, lower zero-shear viscosity favors the direct formation of droplets within the packing zone. This is because higher zero-shear viscosity increases the shear stress of the polymer solution, making it more prone to breakup. At a low rotational speed of 500 rpm, higher zero-shear viscosity also results in the formation of thicker liquid films as the polymer solution flows through the packing zone, again due to increased shear stress. Overall, the zero-shear viscosity has a relatively minor effect on the overall flow pattern distribution; its primary influence lies in the mode of flow pattern formation. Higher zero-shear viscosity leads to greater liquid holdup in the packing zone, easier breakup of the liquid flow, and the formation of thicker liquid films.

#### 3.1.3. Effect of Inlet Velocity

**Figure 9:**
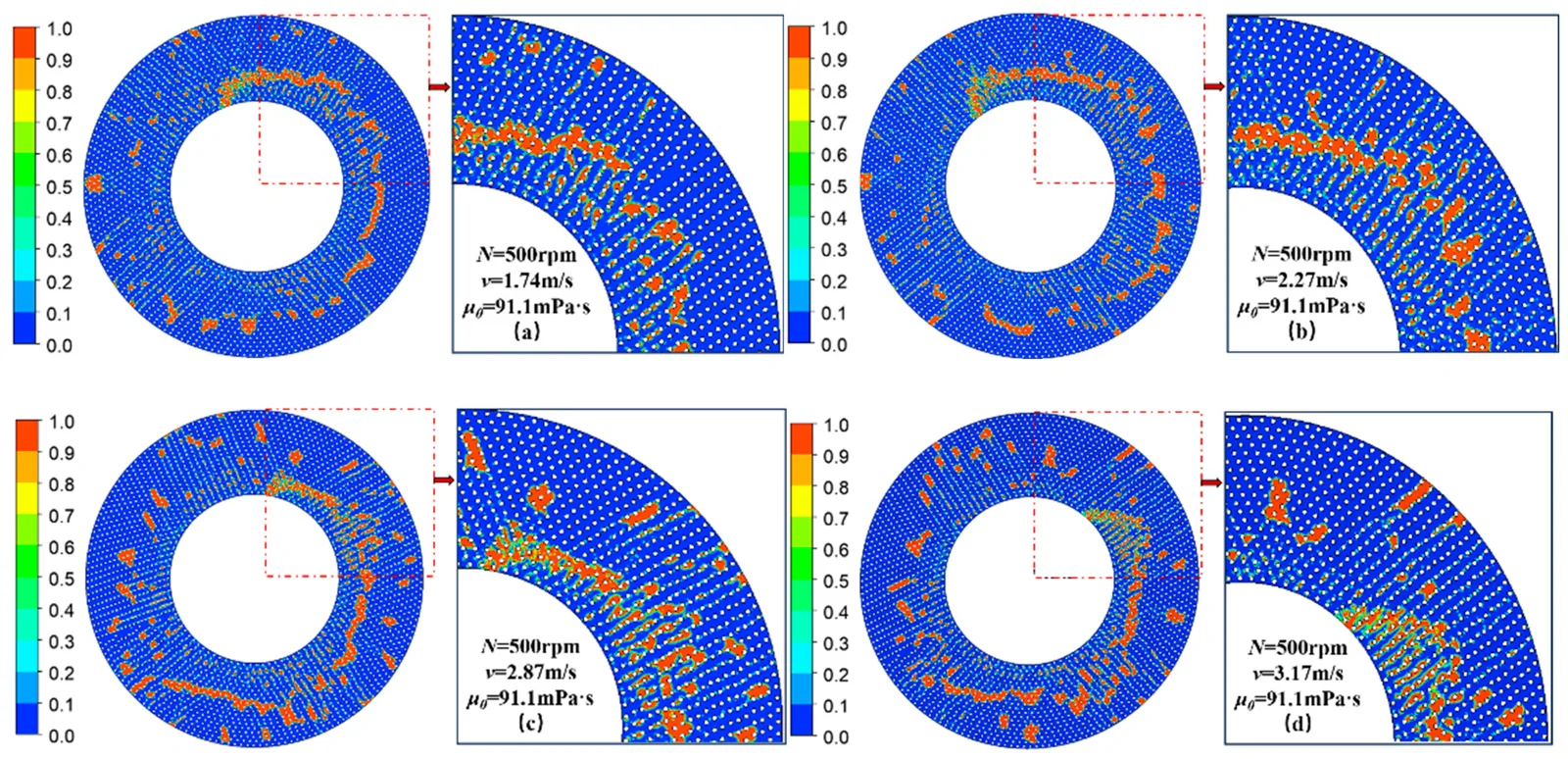
Polymer solution flow patterns in the RPB under different inlet flow velocities. (**a**) *v* = 1.74 m/s; (**b**) *v* = 2.27 m/s; (**c**) *v* = 2.87 m/s; (**d**) *v* = 3.17 m/s.

Figure 9 illustrates the effect of inlet velocity on the flow pattern of the polymer solution in the RPB. It is evident that the total volume of liquid observed in the packing zone increases with increasing inlet velocity. Comparative analysis of the liquid distribution within the packing zone reveals that at higher inlet velocities, the uniformity of liquid distribution on the packing is improved. This can be attributed to the fact that a higher inlet velocity results in a greater liquid flow rate entering the packing zone per unit time, thereby increasing the number of observable liquid elements. Additionally, the increase in inlet velocity enhances the surface renewal rate and turbulent intensity of the liquid phase. Under the action of the high-gravity field, the liquid is more readily broken up, leading to the presence of a greater number of finer liquid elements in the packing zone.

**Figure 10:**
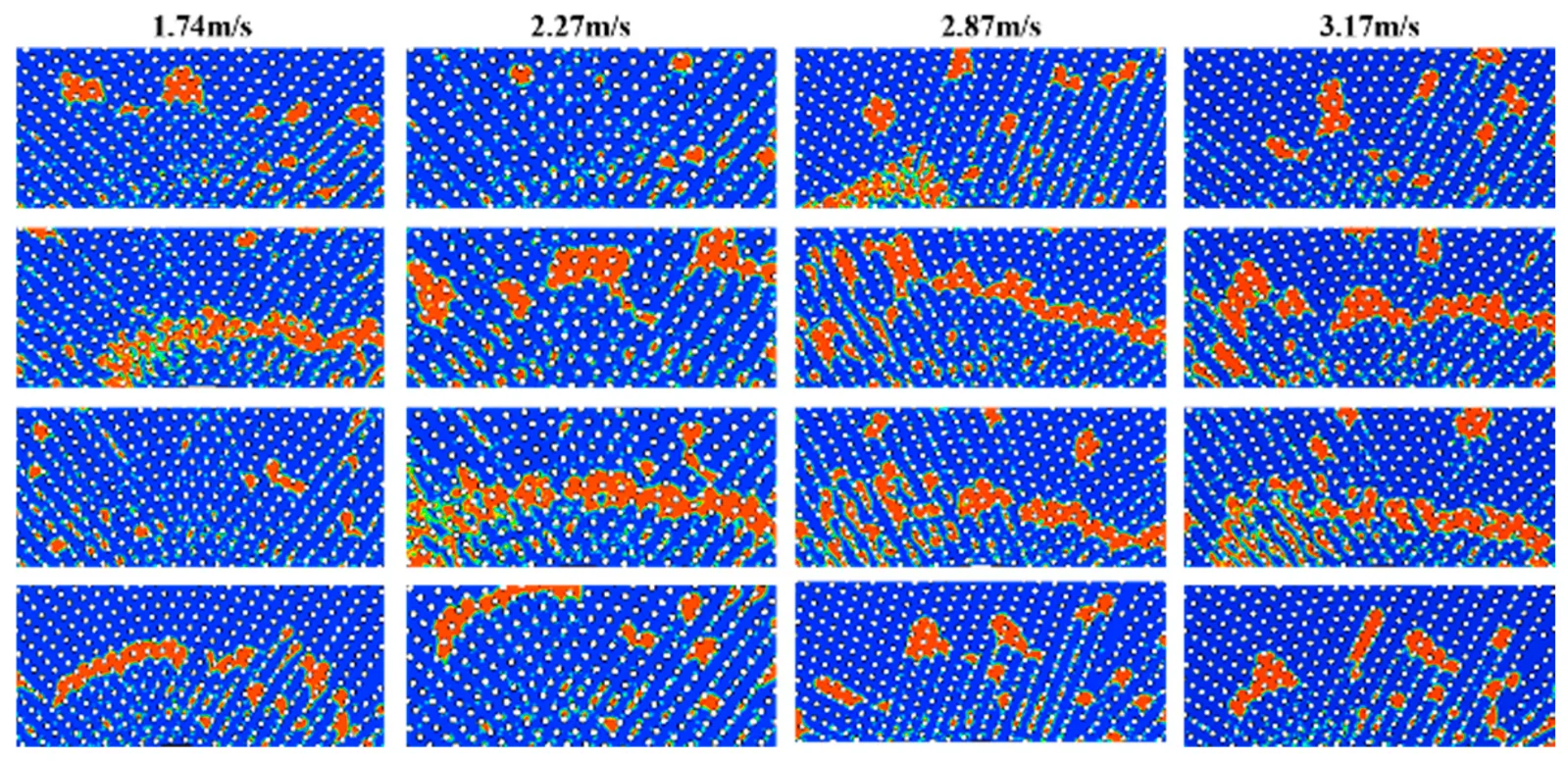
Flow pattern diagrams in packing under different inlet flow velocities.

To further investigate the underlying mechanism, Figure 10 presents a detailed analysis of the flow patterns within the inlet velocity range of 1.74 to 3.17 m/s. At a velocity of 1.74 m/s, droplet flow is the dominant mode, with only a small number of liquid films observed on the packing surface. When the velocity increases to 2.27 m/s, droplet flow remains the primary mode, but both the number and size of droplets increase. Notably, when the velocity reaches 2.87 m/s, a transition in the flow pattern occurs: liquid film flow becomes the dominant mode, and both the number and size of liquid elements increase significantly compared to lower velocities. In summary, with increasing inlet velocity, both the liquid coverage on the packing surface and the size of individual liquid elements tend to increase, and the uniformity of liquid distribution is improved. Specifically, at a low velocity of 1.74 m/s, the flow is dominated by discrete droplets; at moderate velocities of 2.27 to 2.87 m/s, the number and size of droplets increase, and continuous liquid films begin to appear; at a high velocity of 3.17 m/s, the flow pattern transitions to one dominated by continuous liquid films. These systematic changes indicate that increasing the inlet velocity promotes a transition in the flow pattern from a discrete state to a continuous state.

### 3.2. Distribution Characteristics of Apparent Viscosity

#### 3.2.1. Effect of Rotational Speed

**Figure 11:**
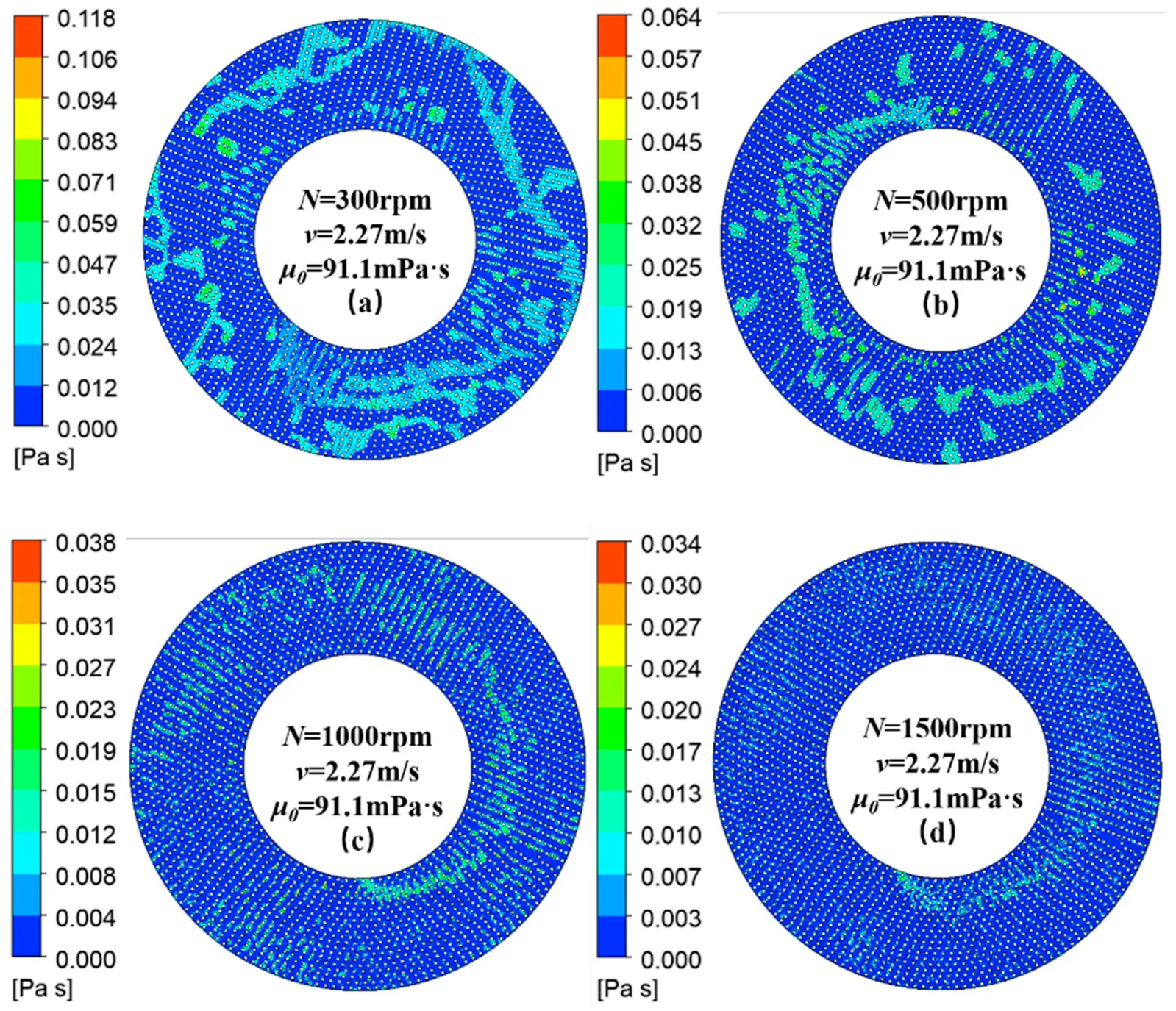
Viscosity variation of polymer solution in RPB under different rotational speeds. (**a**) 300 rpm; (**b**) 500 rpm; (**c**) 1000 rpm; (**d**) 1500 rpm.

Figure 11 presents the spatial distribution of apparent viscosity of the polymer solution within the reactor at different rotational speeds. As shown in the figure, the apparent viscosity of the polymer solution decreases rapidly after being sheared by the packing, and then increases again as the liquid splits into droplets, ligaments, and liquid lumps. Among these, the apparent viscosity of liquid lumps and droplets is higher than that of unstable liquid ligaments, and both exhibit a higher viscosity at the center and lower viscosity at the periphery. This is because, under shear, the irregular shape of the liquid results in a lower shear rate at the central region, leading to a higher apparent viscosity in the core of the liquid elements. With increasing rotational speed, the shear rate increases, resulting in a lower apparent viscosity of the formed liquid elements. At low rotational speeds of 300 to 500 rpm, the maximum apparent viscosity of the liquid within the packing is approximately one-tenth of the zero-shear viscosity. Only a portion of the liquid approaches the minimum shear viscosity, making it prone to forming continuous liquid phases under the influence of viscosity. Consequently, the average diameter of liquid elements is relatively large, and the viscosity distribution is non-uniform. At high rotational speeds of 1000 to 1500 rpm, most of the liquid within the packing exhibits an apparent viscosity close to the minimum shear viscosity, and the maximum apparent viscosity is only about one-thirtieth of the zero-shear viscosity. Under these conditions, the liquid tends not to form continuous phases but instead disperses into finer droplets or smaller liquid lumps, resulting in a smaller average diameter of liquid elements and a more uniform viscosity distribution. Therefore, with increasing rotational speed, the apparent viscosity of the liquid undergoes more drastic changes, and a turning point is observed at approximately 1000 rpm. This is because excessively high rotational speeds cause the apparent viscosity to approach the limit of shear-thinning, beyond which further increases in rotational speed exert a weaker regulatory effect on the viscosity distribution.

#### 3.2.2. Effect of Zero-Shear Viscosity

**Figure 12:**
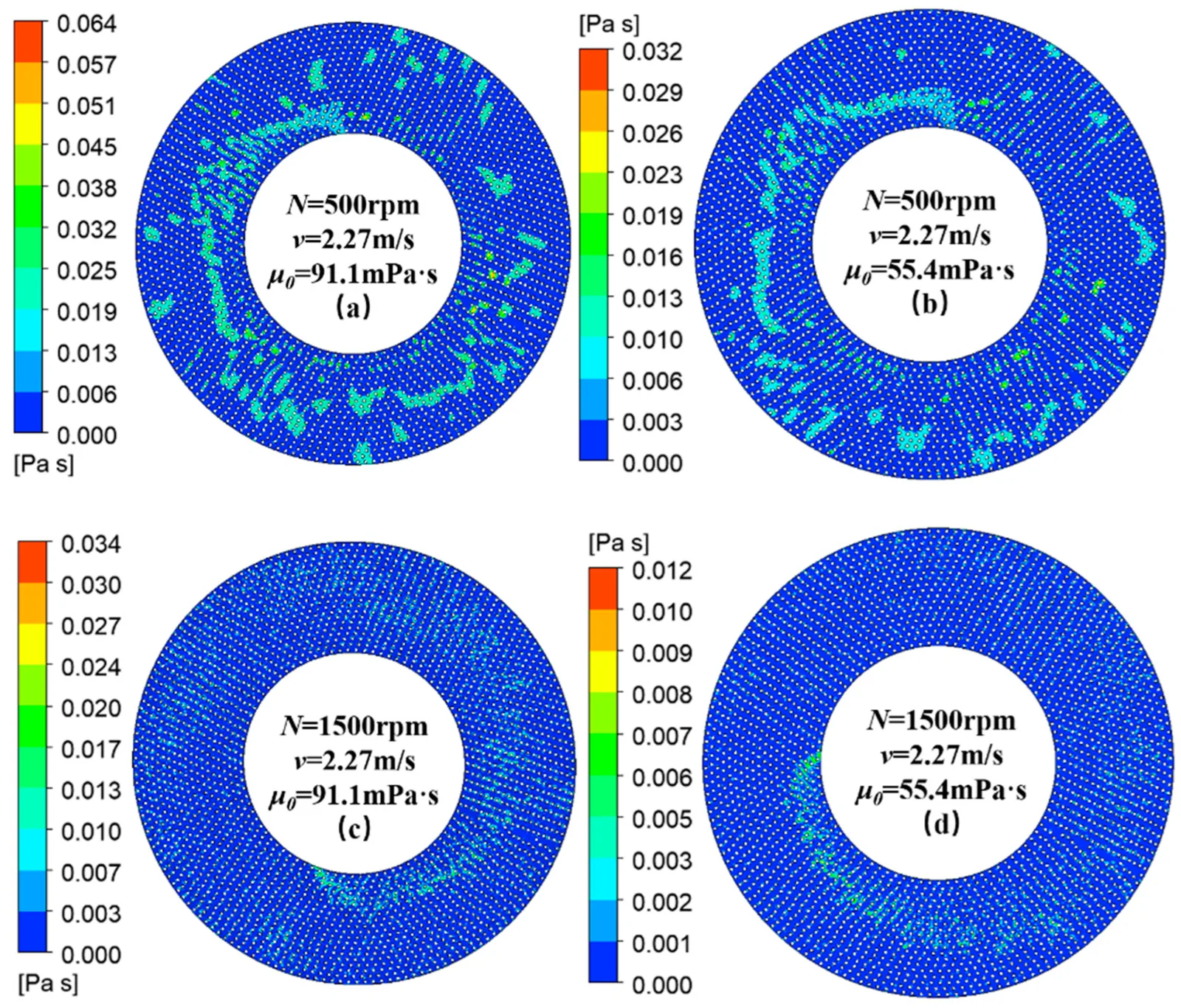
Viscosity variation of polymer solution in RPB under different initial viscosities. (**a**) *N* = 500 rpm, *μ* = 91.1 mPa·s; (**b**) *N* = 500 rpm, *μ* = 55.4 mPa·s; (**c**) *N* = 1500 rpm, *μ* = 91.1 mPa·s; (**d**) *N* = 1500 rpm, *μ* = 55.4 mPa·s.

Figure 12 illustrates the spatial distribution characteristics of apparent viscosity of the polymer solution within the reactor under different zero-shear viscosity conditions. As shown in the figure, under the same shear rate, a higher zero-shear viscosity results in a higher apparent viscosity of the liquid elements formed after shearing, and the liquid tends to aggregate more readily, forming larger liquid lumps or irregular agglomerates. With increasing zero-shear viscosity, the shape of the liquid elements becomes more irregular, the viscosity gradient between the inner and outer edges of the packing increases significantly, and the overall uniformity of viscosity distribution deteriorates. This phenomenon can be attributed to the stronger viscous resistance associated with higher zero-shear viscosity, which not only reduces the liquid flow velocity in the packing zone but also prolongs the liquid residence time, thereby exacerbating local aggregation effects. In addition, due to the differences in shear action at different positions, the shear history within the high-viscosity liquid elements is non-uniform, leading to greater variation in apparent viscosity distribution and more irregular morphology of the droplets or liquid lumps.

During a high-gravity-enhanced polymer dissolution process, the high-speed rotation of the packing provides an intense shear environment, with one of its core objectives being to reduce the apparent viscosity of the polymer through shear action, thereby promoting solvent diffusion and mixing into the polymer and accelerating the swelling and dissolution processes. However, the above analysis indicates that at a high rotational speed of 1500 rpm, the effect of zero-shear viscosity on the spatial distribution of apparent viscosity is not significant. This is mainly because the high shear rate has already brought the apparent viscosity close to the lower limit of shear-thinning, where further variations in zero-shear viscosity have only a limited regulatory effect on the final viscosity distribution. In other words, at high rotational speeds, shear action dominates, and the influence of zero-shear viscosity is substantially weakened; the liquid tends to exhibit a relatively low and uniform apparent viscosity distribution regardless of the zero-shear viscosity. Therefore, the effect of zero-shear viscosity on the spatial distribution of apparent viscosity is more pronounced at low rotational speeds, while it becomes considerably less significant at high rotational speeds. This suggests that in high-gravity-enhanced dissolution processes, appropriately increasing the rotational speed can effectively mitigate the adverse effects of zero-shear viscosity on flow and mixing uniformity, facilitating rapid and uniform dispersion and dissolution of the polymer solution.

#### 3.2.3. Effect of Inlet Velocity

**Figure 13:**
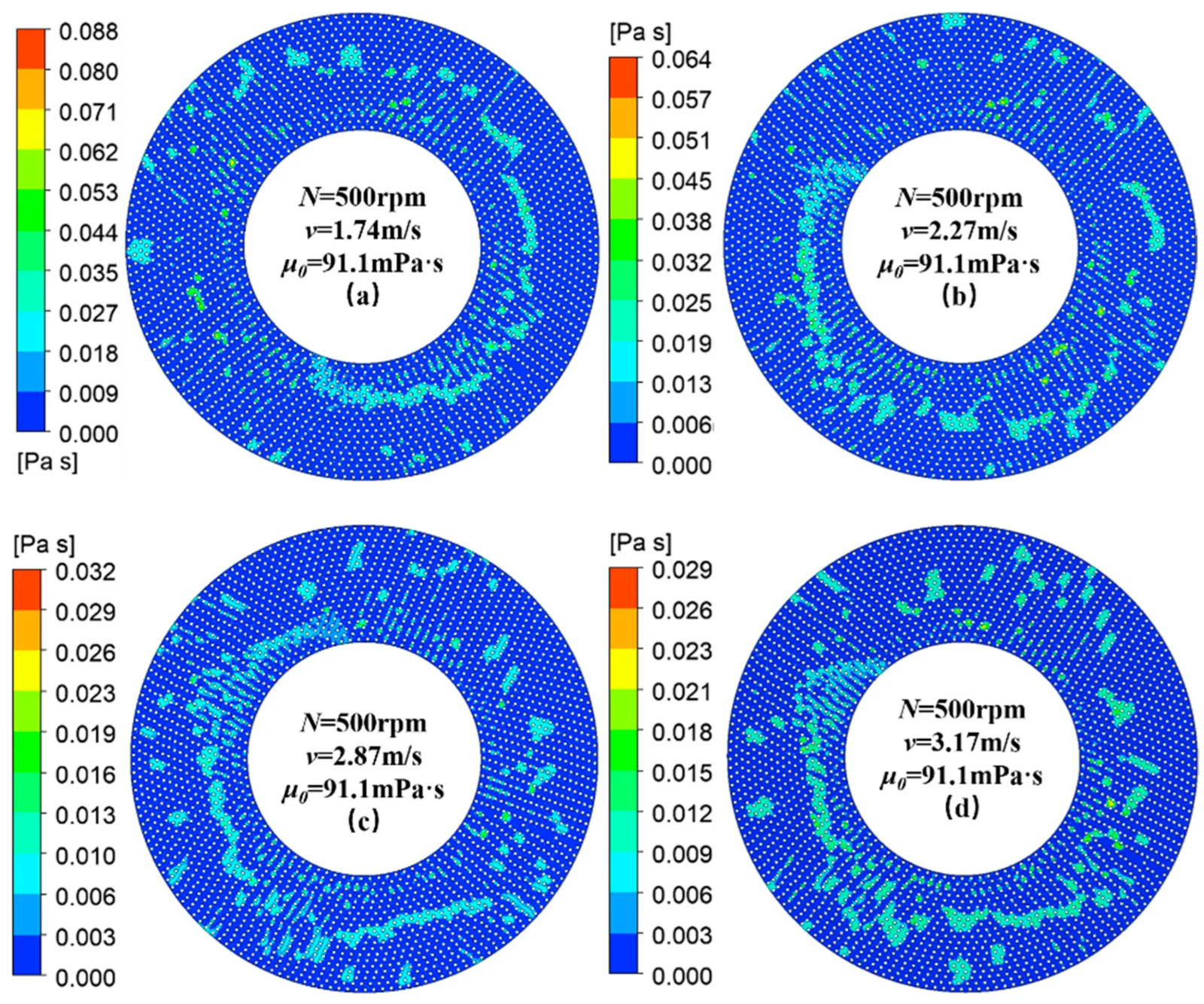
Viscosity variation of polymer solution in RPB under different inlet flow velocities. (**a**) *v* = 1.74 m/s; (**b**) *v* = 2.27 m/s; (**c**) *v* = 2.87 m/s; (**d**) *v* = 3.17 m/s.

Figure 13 illustrates the influence of inlet velocity on the spatial distribution of apparent viscosity of the polymer solution in the RPB. It can be observed that when the inlet velocity varies within the range of 1.74 to 3.17 m/s, the axial distribution of apparent viscosity remains relatively uniform without significant variation. In the radial direction, however, a distinct pattern is evident: the apparent viscosity is higher in the central region and lower in the outer region. This trend arises because, in the outer region of the packing, the polymer solution is thoroughly sheared and dispersed by the high-speed rotating packing, resulting in a significant reduction in liquid micro-element size and a higher degree of dispersion, which in turn lowers the apparent viscosity in this area. In contrast, the central region experiences weaker shear effects and longer liquid residence times, favoring the formation of local high-viscosity zones.

It is worth noting that at an inlet velocity of 2.27 m/s, the axial distribution of apparent viscosity exhibits some fluctuation, which may be attributed to enhanced flow instability under this specific condition. Nevertheless, the radial distribution pattern remains consistent across all inlet velocities tested, with the lowest apparent viscosity consistently observed in the outer region and the highest in the central region. In general, increasing the inlet velocity generally promotes liquid dispersion and surface renewal in the packing zone, thereby enhancing mass transfer efficiency and dissolution rate. However, the present results indicate that the inlet velocity has a limited effect on the spatial distribution of apparent viscosity, particularly in the outer region where the liquid is already nearly fully dispersed and viscosity changes are minimal. This finding suggests that, within the high-gravity field, the shear action of the packing is the dominant factor governing viscosity variation and liquid dispersion, while the regulatory effect of inlet velocity is relatively limited. Consequently, optimizing the rotational speed and packing structure may be more effective in improving polymer dissolution efficiency than simply increasing the inlet velocity.

## 4. Conclusions

In this study, a CFD numerical simulation approach was employed to systematically investigate the flow pattern distribution characteristics of the polymer solution within the packing zone of a high-gravity RPB and their dependence on operating conditions. In view of the shear-thinning behavior of the polymer solution as a non-Newtonian fluid, a two-dimensional computational model was developed by incorporating the VOF multiphase model and the Carreau–Yassuda viscosity model, accounting for viscosity variation and simplifying the wire mesh packing geometry. The effects of rotor rotational speed, inlet velocity, and initial solution viscosity on the flow state, aggregation behavior, and spatial distribution of apparent viscosity were systematically examined. The main conclusions are as follows:

(1) The flow of the polymer solution within the packing zone exhibits pronounced shear-stratified characteristics. With increasing rotational speed, the centrifugal acceleration intensifies, leading to a higher breakup frequency of the liquid film and a gradual transition of the flow pattern from film-dominated to droplet-dominated. However, an increase in zero-shear viscosity suppresses droplet dispersion, resulting in a coexisting flow pattern comprising both liquid films and droplets. In contrast, increasing the inlet velocity promotes the transition from discrete droplet flow to continuous liquid film flow.
(2) Increasing the rotational speed significantly enhances the shear rate, which reduces the apparent viscosity of the solution while improving the uniformity of viscosity distribution. A turning point is observed at approximately 1000 rpm, beyond which the viscosity approaches the lower limit of shear-thinning, and further increases in rotational speed exert a diminishing regulatory effect on viscosity distribution. Higher zero-shear viscosity promotes liquid aggregation, increases the irregularity of droplets or liquid lumps, enlarges the viscosity gradient between the central and outer regions of the packing, and leads to a less uniform viscosity distribution. Within the inlet velocity range of 1.74 to 3.17 m/s, the influence on viscosity distribution is relatively limited; the axial distribution of apparent viscosity remains fairly uniform, whereas the radial distribution shows higher values in the central region and lower values in the outer region.
(3) By incorporating the Carreau–Yassuda viscosity model, the limitations of the conventional VOF model in capturing interfaces in viscous fluid flows were effectively overcome, revealing a shear effect in the polymer solution within the RPB. High rotational speeds can mitigate the adverse effects of elevated zero-shear viscosity, while the synergistic combination of high rotational speed and high inlet velocity may further enhance liquid dispersion.

Future work will extend the present two-dimensional inelastic model to three-dimensional viscoelastic simulations, incorporate polydisperse droplets and multi-droplet impact sequences to better represent real spray conditions, and couple the flow model with mass transfer equations to directly predict polymer dissolution rates. In addition, the effects of the centrifugal force field will be included to bridge the gap between the simplified static conditions and the actual high-gravity environment.

## Data Availability

The data that support the findings of this study are available from the corresponding author upon reasonable request.

## Abbreviations

Greek symbols	
*α*	transition coefficient
α_l_	Volume fraction of liquid phase
α_g_	Volume fraction of gas phase
*ε_L_*	Liquid holdup
ρ	Density, kg/m^3^
σ	Surface tension coefficient, N/m
η_0_	Zero-shear Newtonian viscosity, Pa·s
η_∞_	Limiting viscosity at infinite shear rate, Pa·s
λ	Relaxation time, s
Subscript	
g	Gas phase
l	Liquid phase
Abbreviation	
RPB	Rotating packed bed
CFD	Computational Fluid Dynamics
CSF	Continuum surface force
HPAM	Partially hydrolyzed polyacrylamide
UDF	User-defined function
VOF	Volume of fluid
rpm	Revolutions Per Minute
